# MiR-145-5p Inhibits the Invasion of Prostate Cancer and Induces Apoptosis by Inhibiting WIP1

**DOI:** 10.1155/2021/4412705

**Published:** 2021-12-02

**Authors:** Jianming Sun, Linggang Deng, Ye Gong

**Affiliations:** Department of Urology Surgery, Chenzhou No. 1 People's Hospital, Chenzhou, Hunan 423000, China

## Abstract

Prostate cancer (PCa) is a common malignant tumor of the male genitourinary system that seriously affects the quality of life of patients. Studying the pathogenesis and therapeutic targets of PCa is important. In this study, we investigated the role of miR-145-5p in PCa and its potential molecular mechanisms. The expression levels of miR-145-5p in PCa tissues and adjacent control tissues were detected by real-time quantitative polymerase chain reaction. The effects of miR-145-5p overexpression on PCa were studied using cell proliferation, migration, and invasion experiments. Furthermore, WIP1 was the target gene of miR-145-5p through the bioinformatics website and dual-luciferase reporter gene experiment. Further studies found that WIP1 downregulation could inhibit the proliferation, invasion, and cloning of PCa cells. Overexpression of WIP1 reversed the anticancer effects of miR-145. The anticancer effect of miR-145 was achieved by inhibiting the PI3K/AKT signaling pathway and upregulating ChK2 and p-p38MAPK. Taken together, these results confirmed that miR-145-5p inhibited the growth and metastasis of PCa cells by inhibiting the expression of proto-oncogene WIP1, thereby playing a role in tumor suppression in PCa and may become a potential therapeutic target for the treatment of PCa.

## 1. Introduction 

Prostate cancer (PCa) is one of the most common malignancies of the male genitourinary system. The incidence of PCa ranks first among male malignant tumors, and the mortality rate is second only to lung cancer [1–4]. Currently, many studies are conducted on miRNA and tumors, especially PCa; such studies considerably help in the early diagnosis and prognosis of PCa and provide new targets and ideas for determining the causes of tumor occurrence and its treatment [5–11].

MiRNAs are a class of single-stranded noncoding RNAs with regulatory functions ranging from 20 nt to 24 nt in length [12–14]. miRNAs are mainly involved in the regulation of posttranscriptional levels of mRNAs [15, 16]. The expression profile of miRNA changes in tumors, and many differentially expressed miRNAs are found in PCa [17]. These miRNAs often act on the key pathways of carcinogenesis and promote the formation of PCa by inhibiting apoptosis [18, 19], but some miRNAs also play a role as tumor suppressor genes [20]. miRNA expression may have significant differences in tumor pathological grades or stages. [21]. Formosa et al. [22] believed that methylation silencing of miRNA via promoter CpG island may be an important mechanism of PCa pathogenesis, and the overexpression or reduction of different miRNAs play a regulatory role in the occurrence and development of PCa. miR-145 is an important member of noncoding small RNA molecules [23] located in the human chromosome 9q31 [24]. miR-145 is abnormally expressed in colorectal cancer, lung cancer, gastric cancer, pancreatic cancer, and other solid tumors and plays the role as tumor suppressor genes [25–28]. miR-145 can regulate tumor proliferation through the action of multiple target genes, such as KRAS, c-myc, and FSCN1 [29]. However, studies on miR-145 in PCa are rare.

Wild-type-p53-induced phosphatase 1 (WIP1) gene is a newly discovered proto-oncogene that belongs to a serine/threonine protein phosphatase [30, 31]. WIP1 has a synergistic effect with other oncogenes and was closely related to the occurrence and development of various human malignant tumors [32]. WIP1 is overexpressed in a variety of tumors, including neuroblastoma, breast cancer, ovarian cancer, pancreatic cancer, PCa, liver cancer, gastric cancer, colorectal cancer, thyroid cancer, medulloblastoma, and other tumors; also, WIP1 is closely related to the occurrence and development of breast, gastric, and pancreatic cancers [33]. However, its role in the pathogenesis and regulatory mechanisms of PCa remains unclear [34, 35].

Bioinformatics analysis showed that WIP1 is one of the functional potential target genes of miR-145-5p. WIP1 is a new oncogene upregulated in a variety of tumors, and WIP1 downregulation can inhibit the invasion and metastasis of cancer cells. However, whether miR-145-5p-targeted WIP1 is involved in PCa progression remains unknown. In this study, the effects of miR-145-5p targeted regulation of WIP1 on the proliferation and apoptosis of PCa cells were observed, and the possible mechanisms were discussed.

## 2. Methods

### 2.1. Patient Information and Sample Collection

Cancer tissues and adjacent tissues of 20 patients with pathologically diagnosed PCa in Chenzhou First People's Hospital from July 2016 to June 2019 were collected. The specimens were quickly placed in a −80°C refrigerator for subsequent detection. All specimens were stained using hematoxylin and eosin (HE) in the department of pathology of our hospital before the experiment and were confirmed cancerous or paracancerous benign tissues. The patients had no history of long-term endocrine therapy and chemoradiotherapy before surgery. Patients with other malignant tumors, immune diseases, metabolic diseases, and incomplete clinical data were excluded. The patients were 47–82 years old, with an average age of (64.15 ± 6.14) years. The TNM staging criteria for AJCC PCa in 2010 were used for pathological diagnosis. All patients signed informed consent, and the study was approved by the ethics committee of Chenzhou First People's Hospital.

### 2.2. Cell Culture and Transfection

Human PCa cell lines (LNCaP, DU145, PC3), human prostatic hyperplasia cells (BPH-1), transgenic adenocarcinoma mouse prostate cell (TRAMP-C2), and prostatic epithelial cell line (RWPE-1) were purchased from the American Type Culture Collection (ATCC, Manassas, VA, USA). The above cells were supplemented with RPMI 1640 medium containing 10% fetal bovine serum (Gibco, USA) at 37°C, and the medium was changed once a day. Has-miR-145-5p mimics, mimic-NC, si-WIP1, si-NC, vector-NC, and vector WIP1 were purchased from Guangzhou Ruibo Biology Co., Ltd. When the cells were cultured to 60% fusion degree, the Lipofectamine^TM^ 2000 (Life Technologies, USA) specification was used for transfection of siRNA (50 nmol/L) with liposome Lipofectamine^TM^2000. After 24 h of transfection, the cells were collected for cell proliferation activity detection.

### 2.3. MTT Assay

Cell proliferation ability was determined using the MTT assay. Tumor cells at the logarithmic growth stage were inoculated in 96-well plates. Each well was inoculated with 5 × 10^3^ cells and cultured in the cell incubator for 24 h. The cells were grouped according to experimental purposes. After treatment, the incubation period was continued for 72 h. Exactly 20 *μ*L (5 mg/mL) of MTT solution (Beyotime, Shanghai, China) was added to each well, and the culture medium was removed after 4 h. Then, 150 *μ*L of dimethyl sulfoxide was added to each group. Absorbance was determined at 450 nm by using a microplate reader, and the cell survival rate was calculated. Each group of cells was provided with five repeating wells.

### 2.4. Transwell Invasion Assay

After transfection for 24 h, PCa cells were digested and resuspended in a serum-free RPMI 1640 culture medium. Matrigel (BD, USA) was obtained from the −20°C refrigerator. Matrigel was placed in a refrigerator at 4°C overnight, freeze-thawed, and set aside. Matrigel and the culture medium were mixed at a rate of 1 : 7 and evenly coated on Transwell vesicles (50 *μ*L/hole). Matrigel was solidified in a 37°C cell incubator. The resuspended cells were placed in the upper chamber of Transwell (Millipore, USA). After 24 h, the cells were fixed with a mixture of formaldehyde and acetic acid for 15 min and washed with PBS. The cells that did not pass through the ventricular membrane were wiped off with cotton swabs. Crystal violet was used for staining and then washed with PBS. Three fields were randomly selected for image capturing and counting, and the average number of cells invaded in each field was calculated.

### 2.5. Scratch Closure Migration Assay

Cells during the logarithmic growth stage were collected and made into a single-cell suspension, which was inoculated in a six-well plate with 1 × 10^6^ cells per well. Tumor cells in the logarithmic growth stage were taken and infected when cell confluence reached 80%–90%. The blank control group was not treated. Each group was provided with five multiple holes. After 12 h, a line was drawn using a white 10 *μ*L spear tip. During the scratch experiment, the cell culture medium does not contain serum. After 48 h, ImagePlus software (V 6.0) was used to measure the migration distance between cell scratches. Each experiment was repeated three times.

### 2.6. Flow Cytometry

Cisplatin (10 *μ*M) was used to induce cell apoptosis, and the effect of miR145-5p and WIP1 on apoptosis was detected. PC3 and LNCaP cells were collected after transfection and digested into 1 × 10^5^/mL single-cell suspension. Annexin V-FITC dye was added and mixed. The samples were incubated in the dark for 10 min at room temperature. PI dye was added and incubated at room temperature in the dark for 5 min. The stained cells were detected by flow cytometry.

### 2.7. Western Blot Analysis

Tumor cells were collected, and intracellular proteins were extracted using RIPA lysate (Solarbio, Beijing, China). The BCA kit was used to determine the protein content of the samples. After protein quantification, protein 70 *μ*g was obtained from each group and added to the loading buffer. The protein was denatured at 95°C for 10 min. Electrophoresis was carried out, the membrane was transferred (Millipore, Bedford, MA, USA), and 5% skim milk was sealed. The following primary antibodies were added: PI3K (Abcam, 1 : 1000), p-PI3K (Abcam, 1 : 1000), AKT (Abcam, 1 : 1000), p-AKT (Abcam, 1 : 1000), ChK2 (Abcam, 1 : 1000), p38MAPK (Abcam, 1 : 1000), p-p38MAPK (Abcam, 1 : 1000), Bax (Abcam, 1 : 1000), Bcl-2 (Abcam, 1 : 1000), Cleaved Caspase 3 (Abcam, 1 : 1000), and GAPDH (Abcam, 1 : 1000), and the samples were incubated at 4°C overnight. The secondary antibody (horseradish peroxidase-labeled sheep anti-rabbit, Abcam, 1 : 5000) was added and incubated at room temperature for 2 h. The images were then taken with a gel imaging system after a chemical illuminator was added. The relative protein content was represented by the target protein GAPDH.

### 2.8. Double Luciferase Reporter Gene Assay

TargetScan was used to predict the relationship between WIP1 and miR-145-5p. The 3′-UTR fragment of WIP1 mRNA containing the miR-145-5p binding site and the 3′-UTR fragment of WIP1 mRNA mutated at the miR-145-5p binding site were cloned into the reporter vector pmiR-reporter luciferase vector (Shanghai GenePharma Biological Company, China), and the recombinant plasmids were named WIP1-WT and WIP1-MUT, respectively. Cells from the logarithmic growth stage were collected and inoculated in 6-well plates with 1 × 10^6^/well. When the cells reached 90% fusion degree, miR-145-5p mimics and mimic-NC were co-transfected with the aforementioned recombinant plasmids according to the specification of Lipofectamine™ 2000. Cells were lysed at room temperature for 10 min according to the instructions of the dual-luciferase reporter gene detection kit (Promega, Madison, WI, USA), and then 50 firefly luciferase detection reagent was added. After the samples were mixed well, the relative light unit (RLU) was detected using a fluorescence meter. After an interval of 10 min, 100 *μ*L of Renilla luciferase detection reagent was added, the RLU of the internal reference plasmid pRL-TK was determined after mixing, and relative luciferase activity was calculated.

### 2.9. Real-Time Quantitative Polymerase Chain Reaction (qRT-PCR)

Total RNA was extracted from the cells according to the instructions of TRIzol kit (Beyotime, Shanghai, China) and reverse-transcribed into cDNA. The real-time fluorescence quantitative PCR reaction system was as follows: 1 *μ*L of cDNA, 1 *μ*L of primer, 10 *μ*L of 2 × SYBRGreen Master Mix, and 7 *μ*L of sterile distilled water. The PCR primer sequence was as follows: the upstream sequence of GAPDH was 5′-TGAAGGTCGGAGTCAACGGG-3′, and the downstream sequence was 5′-CCTGGAAGATGGTGATGGG-3′. The upstream sequence of U6 snRNA was 5′-CTCGCTTCGGCAGCACA-3′, and the downstream sequence was 5′-AACGCTTCACGAATTTGCGT-3′. The upstream sequence of WIP1 was 5′-GCCAGAACTTCCCAAGGAAAG-3′, and the downstream sequence was 5′-GGTTCAGGTGACACCACAAATTC-3′. The upstream sequence of miR‐145‐5p was 5′-CAGGAATCCCTTAGATGCTA-3′, and the downstream sequence was 5′-CCATGACCTCAAGAACAGT-3′. The reaction procedure was as follows: predenaturation at 95°C for 3 min, denaturation at 95°C for 15 s, annealing at 58°C for 30 s, and extension at 72°C for 30 s for a total of 40 cycles. WIP1 mRNA (GAPDH as internal reference) and miR-145-5p (U6 snRNA as internal reference) expression was detected using SYBR Green qPCR, and the relative expression levels of WIP1 mRNA and miR-145-5p were calculated using the 2^−ΔΔ*Ct*^ method.

### 2.10. Statistical Analysis

SPSS 21.0 (SPSS Inc., Chicago, IL, USA) statistical software was used for processing, and the measurement data were represented by mean ± standard deviation. Two-tailed unpaired Student's *t*-test was used to compare the two groups of data, whereas one-way ANOVA followed by Tukey's multiple comparison test was used to compare multiple groups of data. The Pearson correlation was used to analyze the co-expression of miR-145-5p and WIP1. The difference was statistically significant if *P* ≤ 0.05.

## 3. Results

### 3.1. MiR-145-5p Expression Was Downregulated in PCa Tissues

**Figure 1:**
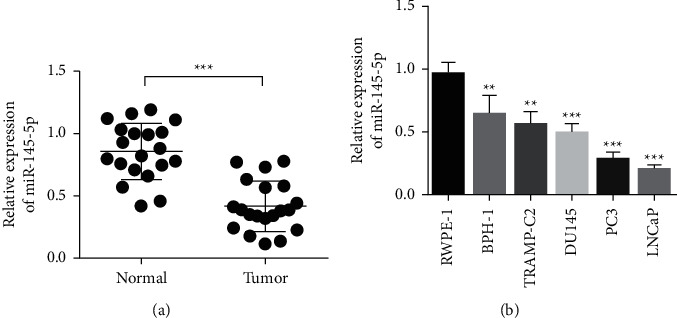
MiR-145-5p is down-regulated in PCa. (a) MiR-145-5p expression level detection. (b) MiR-145-5p expression in human normal prostate epithelial cells (RWPE-1) and PCa cells (BPH-1, LNCaP, DU145, PC3, and TRAMP-C2). ^∗∗^*P* < 0.01 and ^∗∗∗^*P* < 0.001.

To study the role of miR-145-5p in PCa, we measured the expression of miR-145-5p in cancer tissues and the adjacent tissues of 20 patients with PCa. MiR-145-5p expression in PCa tissues was decreased compared with that in the paracancer control group (Figure 1(a)). Then, we measured the expression of miR-145-5p in human normal prostatic epithelial cells (RWPE-1) and PCa cells (BPH-1, LNCaP, DU145, PC3, and TRAMP-C2). Compared with that in RWPE-1 cells, miR-145-5p expression in PCa cells was significantly reduced.  Figure 1(b) experimental results showed that the expression of miR-145 in LNCaP and PC3 was the lowest. Therefore, we investigated the effect of overexpression of miR-145 on the biological behavior of LNCaP and PC3 cells.

### 3.2. Overexpression of miR-145-5p Inhibited the Proliferation, Invasion, and Migration of LNCaP and PC3 Cell Lines

**Figure 2:**
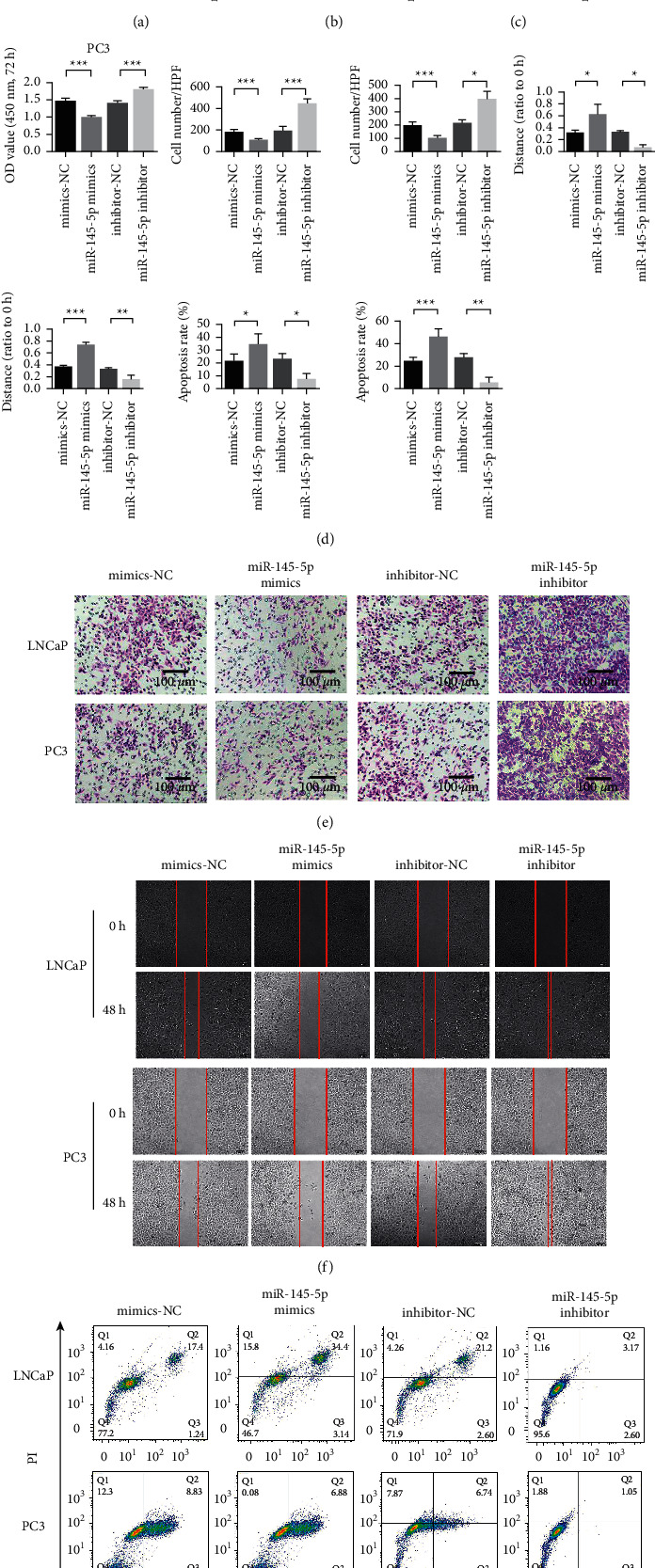
Overexpression of miR-145-5p inhibits the proliferation, invasion and migration of PCa LNCaP and PC3. (a) Detection of miR-145-5p expression efficiency in LNCaP cells. (b) Detection of miR-145-5p expression efficiency in PC3. (c) LNCaP cell proliferation assay. (d) PC3 cell proliferation test. (e) Transwell detection of LNCaP and PC3 cells. (f) LNCaP and PC3 cell scratch test. (g) LNCaP and PC3 cell apoptosis detection by flow cytometry. ^*∗*^*P* < 0.05, ^∗∗^*P* < 0.01, and ^∗∗∗^*P* < 0.001. Magnification 200×.

Next, we studied the effects of miR-145-5p overexpression and inhibition on the proliferation, invasion, metastasis, and apoptosis of LNCaP and PC3 cells. The experiment was divided into four groups named mimic-NC (negative control), miR-145-5p mimics, inhibitor-NC, and miR-145-5p inhibitor. The results of the miR-145-5p expression efficiency test in LNCaP cells showed that miR-145-5p mimics could effectively upregulate miR-145-5p expression, while the miR-145-5p inhibitor could decrease the expression of miR-145-5p (Figure 2(a)). The expression level of miR-145-5p in PC3 was consistent with that in LNCaP cells (Figure 2(b)). Furthermore, the effect of miR-145-5p on the proliferation of LNCaP and PC3 cells was detected using a cell proliferation assay. MiR-145-5p overexpression inhibited the proliferation of PCa cells, while miR-145-5p inhibition promoted the proliferation of PCa cells (Figures 2(c) & 2(d)). Next, we detected the effect of miR-145-5p on the invasion ability of LNCaP and PC3 cells by cell Transwell assay. The results showed that miR-145-5p overexpression inhibited the invasion of PCa cells, while miR-145-5p inhibition promoted the invasion of PCa cells (Figure 2(e)). The effect of miR-145-5p on the migration ability of LNCaP and PC3 cells was detected by the cell scratch test. Overexpression of miR-145-5p inhibited the migration of PCa cells, while inhibition of miR-145-5p promoted the migration of PCa cells (Figure 2(f)). We further examined the effect of miR-145-5p on the apoptosis of LNCaP and PC3 cells through apoptosis experiments. Overexpression of miR-145-5p promoted the apoptosis of PCa cells, while inhibition of miR-145-5p decreased it (Figure 2(g)).

### 3.3. Overexpression of miR-145-5p Inhibited the PI3K/AKT Signaling Pathway and Upregulated the Expression of ChK2 and p-p38MAPK

**Figure 3:**
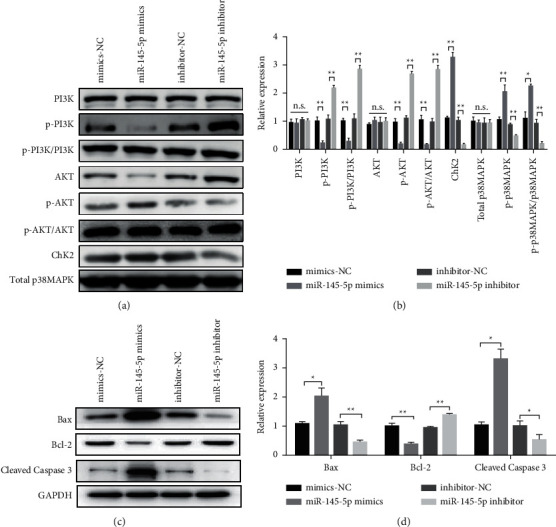
Overexpression of miR-145-5p inhibits the PI3K/AKT signaling pathway and upregulates ChK2 and p-p38MAPK expression. (a) Western blot analysis of PI3K, p-PI3K, AKT, p-AKT, ChK2, Total p38MAPK and p-p38MAPK. MiR-145-5p overexpression had no significant effect on the total PI3K and AKT, while inhibition of the expression of p-PI3K and p-AKT. (b) Statistical analysis results of each group of proteins. (c) Western blot analysis of Bax, Bcl2 and Cleaved Caspase 3. (d) Statistical analysis results of each group of proteins. ^*∗*^*P* < 0.05 and ^∗∗^*P* < 0.01.

To study the molecular mechanism of miR-145-5p affecting the proliferation, invasion, metastasis, and apoptosis of PCa cells, we detected the PI3K/AKT signaling pathway and the expression of ChK2 and p38MAPK. The experiment was divided into four groups, namely, mimic-NC, miR-145-5p mimics, inhibitor-NC, and miR-145-5p inhibitor. MiR-145-5p overexpression had no significant effect on the total PI3K and AKT, while inhibition of the expression of p-PI3K and p-AKT. ChK2 and p-p38MAPK are closely related to cell apoptosis. Further detection results showed that miR-145-5p overexpression could upregulate the expression of ChK2, and overexpression of miR-145-5p could upregulate the expression of p-p38MAPK; this finding suggests that miR-145-5p induced apoptosis of PCa cells by upregulating the expression of the pro-apoptotic protein (Figures 3(a) & 3(b)). In addition, Bax, Bcl2, and Caspase 3 expression was analyzed, and the results showed that miR-145 mimics could upregulate the expression of apoptotic proteins Bax and Caspase 3. The expression of the antiapoptotic protein Bcl-2 was inhibited (Figures 3(c) & 3(d)).

### 3.4. MiR-145-5p Targeted Binding to WIP1

**Figure 4:**
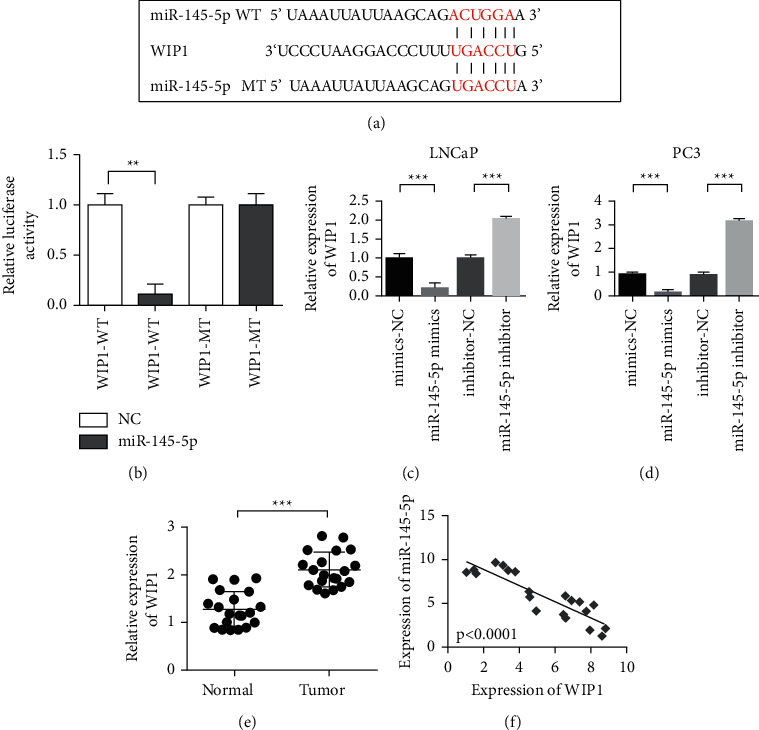
MiR-145-5p targeted binding to WIP1. (a) Information maps of miR-145-5p binding site with WIP1. (b) Dual-luciferase reporter gene demonstrating the binding of miR-145-5p to WIP1. (c) LNCaP cell level showing that miR-145-5p overexpression inhibits WIP1 and inhibits miR-145-5p upregulation of WIP1. (d) PC3 cell level showing that overexpression of miR-145-5p inhibits WIP1 and inhibits miR-145-5p upregulation of WIP1. (e) WIP1 is upregulated in PCa. (f) Detection of the correlation between miR-145-5p and WIP1 co-expression. ^∗∗^*P* < 0.01 and ^∗∗∗^*P* < 0.001.

To study the downstream binding target genes of miR-145-5p, we predicted the downstream binding target genes through the TargetScan website (http://www.targetscan.org/vert_72/). MiR-145 and WIP1 were predicted to have the highest predicted binding score by TargetScan and MiR-145-5p could bind WIP1 (Figure 4(a)). The dual-luciferase reporter gene assay further demonstrated the binding of miR-145-5p to WIP1 (Figure 4(b)). MiR-145-5p mimics had inhibitory activity on the promoter of wild-type WIP1 but not on the mutant WIP1. Furthermore, we detected the effect of miR-145-5p on WIP1. The experimental results showed that miR-145-5p overexpression inhibited the expression of WIP1, while miR-145-5p overexpression inhibited the expression of WIP1 (Figure 4(c)). Detection results of PC3 cells were consistent with that of LNCaP cells. MiR-145-5p overexpression inhibited the expression of WIP1, while miR-145-5p inhibited the upregulated expression of WIP1 (Figure 4(d)). The expression level of WIP1 in prostate cancer showed that compared with the paracancer control group, WIP1 was highly expressed in the tissues of patients with prostate cancer (Figure 4(e)). The correlation between miR-145-5p and WIP1 showed that miR-145-5p expression was negatively correlated with WIP1 (Figure 4(f)).

### 3.5. Knockdown of WIP1 Inhibited the Proliferation and Invasion and Increased Apoptosis of PCa Cells

**Figure 5:**
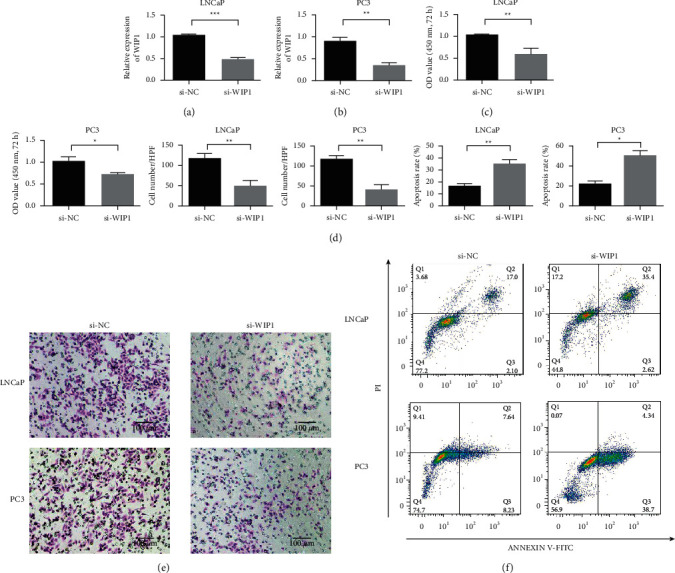
Knockdown of WIP1 inhibited the proliferation and invasion and increased apoptosis of PCa cells. (a) Detection of WIP1 expression in LNCaP cells. (b) Detection of WIP1 expression in PC3 cells. (c) LNCaP cell proliferation assay. (d) PC3 cell proliferation test. (e) Transwell detection of LNCaP and PC3 cells. (f) LNCaP and PC3 apoptosis detection. ^*∗*^*P* < 0.05, ^∗∗^*P* < 0.01, ^∗∗∗^*P* < 0.001, and ^∗∗∗^*P* < 0.001. Magnification 200×.

Studies have reported that WIP1 is related to proliferation [36–39]. Therefore, we used MTT to detect changes in tumor cell proliferation and tumor cell viability. We used siRNA to knock down WIP1 and to study the effect of WIP1. Si-WIP1 could effectively reduce WIP1 expression (Figure 5(a)). Detection results of PC3 cells were consistent with that of LNCaP cells (Figure 5(b)). The effect of WIP1 on the proliferation of PCa cells was detected by MTT. Cell proliferation was inhibited after WIP1 was knocked out (Figures 5(c) & 5(d)). Cell Transwell experiments showed that the invasion ability of cells was reduced without WIP1 (Figures 5(e)). The apoptosis detection results of LNCaP cells showed that apoptosis increased after WIP1 expression was reduced. The results of PC3 cell apoptosis detection were consistent with those of LNCaP cells, and apoptosis increased after WIP1 inhibition (Figure 5(f)).

### 3.6. Overexpression of WIP1 Reversed the Anticancer Effect of miR-145-5p

**Figure 6:**
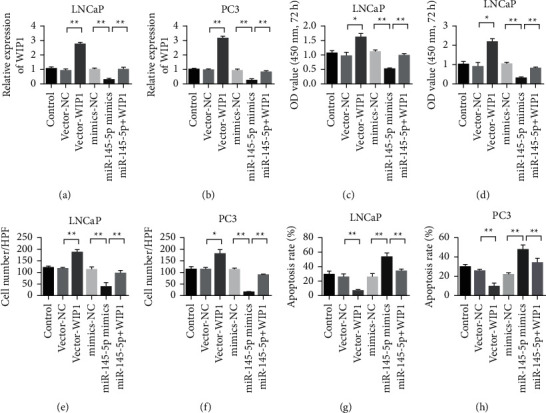
Overexpression of WIP1 reverses the tumor suppressive effect of miR-145-5p. (a) Detection of WIP1 expression in LNCaP cells. (b) Detection of WIP1 expression in PC3 cells. (c) Detection of LNCaP cell proliferation rate. (d) PC3 cell proliferation rate detection. (e) Transwell detection of LNCaP cells. (f) Transwell detection of PC3 cells. (g) LNCaP apoptosis detection. (h) PC3 cell apoptosis detection. ^*∗*^*P* < 0.05 and ^∗∗^*P* < 0.01. Magnification 200×.

To verify the relationship between WIP1 and miR-145-5p, we simultaneously transfected the WIP1 plasmid and miR-145-5p mimics into PCa cells. The expression of WIP1 in LNCaP cells and PC3 showed that the WIP1 overexpressed plasmid could effectively upregulate the expression of WIP1, while miR-145-5p mimics inhibited the expression of WIP1 (Figures 6(a) & 6(b)). The cell proliferation detection showed that WIP1 overexpression promoted the proliferation of PCa cells, miR-145-5p mimics inhibited the proliferation of PCa cells, and WIP1 could reverse the proliferation inhibition of miR-145-5p (Figures 6(c) & 6(d)). The Transwell test results of the cells showed that WIP1 overexpression promoted the invasion ability of PCa cells, miR-145-5p mimics inhibited the invasion ability of PCa cells, and WIP1 could reverse the invasion inhibition ability of miR-145-5p (Figures 6(e) & 6(f)). Apoptosis detection results showed that overexpression of WIP1 reduced apoptosis of PCa cells, miR-145-5p mimics promoted apoptosis of PCa cells, and WIP1 could reverse the apoptotic effect of miR-145-5p (Figures 6(g) & 6(h)).

## 4. Discussion

The 3′-UTR of WIP1 was predicted using TargetScan, and the complementary binding of miR-145-5p was detected. WIP1 may be the direct target gene of miR-145-5p. WIP1 is one of the target genes related to the cell cycle and proliferation.

WIP1 is a newly discovered proto-oncogene, which is overexpressed in a variety of tumors and is closely related to the occurrence, development, and prognosis of tumors [40]. However, its carcinogenic effect has not been fully studied. WIP1 can act on at least seven target proteins, including p53, p38MAPK, ATM, ChK1, ChK2, MDM2, and UNG2. WIP1 is a major factor that inhibits the function of p53 and plays a role in the upstream of p53, which is also one of its major carcinogenic mechanisms. WIP1 can be dephosphorylated and inactivated by p53 protein via p38MAPK, ChK1, and other pathways, reducing its transcriptional activity and playing its carcinogenic role. After the intervention of WIP1 gene, the expression of the downstream pathway or protein is controlled by the WIP1/NF-*κ*B signaling pathway, thus affecting the biological characteristics of the cell, which is believed to be related to NF-*κ*B. WIP1 has also been studied as a target for tumor therapy. Yod et al. [41] silenced the WIP1 of acute promyelocytic leukemia cell lines and found that the apoptosis induced by ChK2 and P38MAPK can be enhanced and improve the sensitivity of leukemia cells to apoptosis [31, 42]. By referring to literatures, we found that the downstream regulatory pathways of WIP1 included PI3K, AKT, ChK2, p-p38MAPK, and so on [39, 43–50]. Therefore, we also selected the above indicators for testing. In the pre-experimental stage, we also detected the expression levels of other proteins, including JNK and STAT3. Among them, only the PI3K/AKT pathway is the most sensitive to miR-145-5p.

In the present study, miR-145-5p expression in PCa cells was upregulated, and the changes in biological characteristics of the transfected tumor cells were observed through cell function experiments in vitro. MiR-145-5p overexpression caused a decrease in cell proliferation and invasion. At the same time, the rate of early apoptosis was increased. In this study, miR-145-5p mimics was used to overexpress miR-145. miR-145 inhibitor was used to reduce the expression of miR-145. In addition, based on the reported literature [51, 52] and our experimental results, we believe that miR-145 has a comprehensive antitumor effect. The antitumor effect of miR-145 is not limited to inducing apoptosis. Transwell and the scratch test confirmed that miR-145 can inhibit the invasion and migration of prostate cancer. Our preliminary experimental results showed that miR-145 had no significant effect on normal bladder cancer cells (Figure S1). WIP1, PI3K/AKT, ChK1, and p38MAPK expression was verified by qRT-PCR and Western blot analysis. miR-145-5p overexpression inhibited mRNA and protein expression of WIP1, accompanied by increased protein expression of ChK1 and p38MAPK. In addition, the antitumor effect of miR-145-5p may also be through phosphatase activity of WIP1 and found the effects on phosphorylation of signaling intermediates.

miR-145-5p can function as a tumor suppressor gene in PCa cells that may be realized by inhibiting WIP1 expression in cells and blocking the pathway of WIP1 to cause cell cycle arrest and cell apoptosis. miR-145-5p may inhibit the proliferation and invasion of PCa cells by regulating the expression of WIP1 and the PI3K/AKT pathway, acting as an oncogene. As one of the miRNAs, miR-145-5p can also act on a variety of target genes. However, WIP1 can be regulated by other miRNAs. In the present work, WIP1 expression was decreased by upregulating miR-145-5p, and changes in the biological characteristics of cells were detected, which is a one-way regulatory mechanism. This study verified that miR-145-5p has the function of regulating WIP1. In further studies, WIP1 should be removed from the experimental level of transgenic animals to further investigate the mechanism of action between miR-145-5p and WIP1 in PCa.

In this study, the regulatory relationship between miR-145-5p and WIP1 was reported for the first time. Although the anticancer effects of miR-145-5p have been reported, this study has revealed newer molecular mechanisms. This study also has some shortcomings. At present, limited to experimental conditions, only one normal prostatic cell line can be obtained. In subsequent studies, if more normal prostate cell lines are available, we will continue to detect the expression level of miR-145.

## 5. Conclusion

In this study, we found that miR-145-5p inhibits the invasion of PCa cells by inhibiting the expression of WIP1. Therefore, elucidating the mechanisms behind miR-145-5p expression may serve as a theoretical foundation for the molecular therapy of PCa in the future. Although significant barriers hinder the clinical application of gene therapy, the discovery of key molecules in the process of cancer development can provide further evidence and strategies.

## Data Availability

The data sets generated during the study are available from the corresponding author on reasonable request.

## Supplementary Materials

Supplementary Materials Figure S1: fffect of miR-145-5p on normal prostatic epithelial cell line (RWPE-1).
