# Supplementary figures and images for: MiR-145-5p Inhibits the Invasion of Prostate Cancer and Induces Apoptosis by Inhibiting WIP1
Source: J Oncol. 2021 Dec 2;2021:4412705. doi: 10.1155/2021/4412705 (PMC8660234; doi:10.1155/2021/4412705)

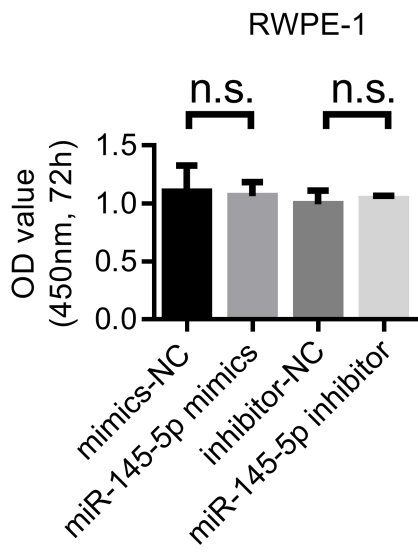

Supplement: Supplementary Materials — Figure S1: fffect of miR-145-5p on normal prostatic epithelial cell line (RWPE-1). [file 4412705.f1.pdf]
